# Acquisition of Knowledge and Practical Skills after a Brief Course of BLS-AED in First-Year Students in Nursing and Physiotherapy at a Spanish University

**DOI:** 10.3390/ijerph16050766

**Published:** 2019-03-03

**Authors:** Carlos Méndez-Martínez, Santiago Martínez-Isasi, Mario García-Suárez, Medea Aglaya De La Peña-Rodríguez, Juan Gómez-Salgado, Daniel Fernández-García

**Affiliations:** 1 Division of Nursing, University Hospital of León, 24008 León, Spain; cmendm00@estudiantes.unileon.es (C.M.-M.); mgarcs06@estudiantes.unileon.es (M.G.-S.); 2 Health and Podiatry Unit, Department of Health Sciences, Faculty of Nursing and Podiatry, Universidade da Coruña, Campus de Esteiro, 15403 Ferrol, Spain; santiago.martinez.isasi@udc.es; 3 San Francisco Hospital, 24004 León, Spain; mpenar00@estudiantes.unileon.es; 4 Department of Nursing, University of Huelva, 21007 Huelva, Spain; 5 Safety and Health Posgrade Program, Espíritu Santo University, Samborondón, Guayaquil 092301, Ecuador; 6 Department of Nursing and Physiotherapy, University of Leon, 24071 León, Spain; dferg@unileon.es

**Keywords:** Cardiopulmonary Resuscitation, CPR, Basic Cardiac Life Support, nursing students, training

## Abstract

Out-of-hospital cardiorespiratory arrest is one of the leading causes of death in the Western world. Early assistance with quality Cardiopulmonary Resuscitation (CPR) and the use of a defibrillator may increase the percentage of survival after this process. The objective of this study was to evaluate the effect of CPR training and the management of an Automatic External Defibrillator (AED). A descriptive, cross-sectional, observational study was carried out among students in the first year of a Nursing and Physiotherapy degree of the University of León. To achieve this goal, a theoretical-practical educational intervention of four hours’ duration which included training on CPR, AED and Basic Life Support (BLS) was carried out. A total of 112 students were included. The results showed an increase in theoretical knowledge on BLS as well as on CPR and AED, and practical skills in CPR and AED management. A theoretical exposition of fifteen minutes and the practical training of CPR wasenough for the students to acquire the necessary theoretical knowledge, although the participants failed to reach quality criteria in CPR. Only 35.6% of students reached the right depth in compressions. Also, ventilation was not performed properly. Based on the results, we cannot determine that the percentage of overall quality of CPR was appropriate, since 57.6% was obtained in this respect and experts establish a value higher than 70% for quality CPR. There was a clear relationship between sex, weight, height and body max index (BMI), and quality CPR performance, being determinant variables to achieve quality parameters. Currently, Basic Life Support training in most universities is based on training methods similar to those used in the action described. The results obtained suggest implementing other training methods that favour the acquisition of quality CPR skills.

## 1. Introduction

Out-of-hospital cardiorespiratory arrest (OHCA) is one of the leading causes of death in Europe, being potentially reversible if treated at an early stage [1]. The percentage of survival of patients in which CPR is conducted immediately after the cardiorespiratory arrest is two to four times higher than that of those who do not receive it, so the start of assistance by witnesses takes on special importance [2]. This survival rate increases when a defibrillator is used in addition to performing CPR, time being a key factor to maximise this percentage [1]. High-quality CPR is of vital importance and is the cornerstone of a series of actions that can reverse the state of OHCA in patients. Quality CPR consists of providing an appropriate frequency and depth of compressions, a correct chest decompression, minimised interruptions, and non-exceeded appropriate volume of ventilations [1]. However, several studies suggest that quality CPR performance is not optimal in most cases, even among healthcare staff that have previously been instructed in this kind of assistance [3,4]. 

The teaching of cardiopulmonary resuscitation is complex as it includes both theoretical knowledge and psychomotor skills. Acquired skills diminish in a span of 3 to 6 months, which implies a need to develop effective learning and retraining strategies [5,6,7,8,9,10].

Among the various methods of CPR training for Health Sciences students, we find some that propose reading for the theoretical training, educational videos on CPR and the defibrillator [7], training in knowledge and skills with its subsequent feedback [5], theoretical lessons taught by an instructor for the acquisition of knowledge, manikins to learn skills [8], high-fidelity simulation, online theoretical lessons and simulation with manikins [11], self-directed teaching, and traditional teaching for retraining groups [12]. However, there is no universally accepted and proven gold standard efficiency method.

There is no consensus about how long the training must be. In the works consulted by the authors, from one up to four hours of training are proposed. The same applies to the period of time after which knowledge and skills should be recycled, ranging from one to six months after the end of the course [2,5,7,8]. The objective of this study was to explore the effect of training in the acquisition of knowledge of Basic Life Support (BLS), CPR skills and the management of Automatic External Defibrillator (AED) in Health Sciences university students.

## 2. Materials and Methods 

### 2.1. Design 

A quasi-experimental, before-after type of study with a control group was conducted among students in their first year of the Nursing Degree and Physiotherapy Degree at the University of León (Spain). The Nursing degree is taught at the campuses of León and Ponferrada, while the Physiotherapy degree is only taught in Ponferrada. The study was developed during the academic year 2017–2018.

### 2.2. Material and Human Resources

For the procedure, 6 standard manikins Resusci Anne (Laerdal®, Stavanger, Norway), two torsos of CPR manikins without feedback, 3 AEDs, and 6 computers with the Resusci Anne Skill Reporter (Laerdal®, Stavanger, Norway) software were used as material resources. Human resources included 6 supervisors, two of them nurses and instructors in Basic Life Support (BLS) by the Spanish Society of Intensive Medicine, Critical Care and Coronary Units (SEMICYUC), two nurses and two nursing students with specific training in CPR. All of them had performed the correct BLS sequence and had obtained a QCPR higher than 95%.

### 2.3. Instrument 

The knowledge questionnaire was used for the pre-test and the post-test. It consisted of 10 items and was prepared ad hoc. For its development, the recommendations on CPR made in 2015 by the European Resuscitation Council (ERC) were consulted. The questionnaire consisted of ten questions with four multiple-choice options, one being correct. The estimated time for completion was 7 minutes. After the questionnaire, a document was delivered with instructions to participants, where data confidentiality was guaranteed.

A standard manikin Resusci Anne (Laerdal®, Stavanger, Norway) was used for the practice and for results measurement. Data were obtained from the CPR Resusci Anne Skill Reporter (Laerdal®, Stavanger, Norway) meter, already employed in similar studies [13,14,15]. The system was programmed using the CPR parameters proposed by the ERC in 2015: 50–60 mm depth of compression, 100–120 compressions per minute of frequency, and 500–600 cm^3^ of inspiratory volume [1]. The information provided by the meter was not shown to the participants during the evaluation. The obtained data were used to create an individual report of CPR of each participant. In addition, the exertion applied by the participants was analysed after their performance by means of the Borg rating of perceived exertion scale (RPE scale) [16].

### 2.4. Variables 

The dependent variables of the study were those derived from the questionnaire regarding the theoretical part and those linked to CPR performance—both the subjective and the objective evaluations—as for the practical part. Table 1 shows the relationship between the variables derived from the questionnaire and those derived from the CPR.

The knowledge questionnaire included independent variables of the pre-test or post-test, date of birth, weight, height, body mass index, belonging to the Campus of León or Ponferrada, and university degree of the participants: Nursing or Physiotherapy.

#### Participants 

The study population was constituted by first-year students of Nursing and Physiotherapy (León and Ponferrada campuses) of the University of León as convenience sampling. The study population included students who were enrolled in the first course of the Nursing and Physiotherapy degree during the study period and who voluntarily decided to participate by signing the informed consent. Those participants who had done some BLS or CPR course in the two years prior to the completion of the action were included. The exclusion criteria were: incorrect, deficient, or incomplete filling of the questionnaire.

A subjective sampling technique by reasoned decision was followed, according to the criteria of qualification and campus of the participants. The sample was considered relevant given its homogeneous nature with respect to the reference population for the criteria of belonging to the same branch of knowledge. It was composed of 3 groups of students (Nursing Degree, Campus of León, Nursing Degree, Campus of Ponferrada, and Physiotherapy Degree, Campus of Ponferrada), obtaining a total of 112 students.

### 2.5. Characteristics of the Action

A theoretical and practical educational action took place, aimed at the training of Health Sciences students of the University of León in BLS and AED operating techniques. The action was dynamic and participatory and was developed from an approach that favoured the students’ motivation and awoke their interest. The training was conducted by following the recommendations of the ERC in its latest guidelines updated in 2015 [1].

The educational action was structured in a single session of 4 h for each group, which was organised as follows:

- A: 20’. Informed verbal consent and pre-test knowledge questionnaire.
- B: 15’. Theoretical lesson.
- C: 60’. BLS sequence practical training (20’), AED practical training (20’), CPR practical training (20’).
- D: 15’. Break.
- E: 120’. Practical evaluation, exertion applied evaluation.
- F: 10’. Post-test knowledge questionnaire.

All students performed a minimum of 5 min of chest compressions and a BLS and AED sequence 5 times.

Both the instructor/student and the manikin/student ratio were 1/6. The manikin/instructor ratio was 1/1.

After the training, the students completed the initial questionnaire and simulated CPR on a manikin for two minutes. The clinical case was the same for all individual tests.

In order to minimise bias, the evaluation was developed by other instructors who were not their coaches. Prior to the training, they were informed about the worksheet and the key points to consider.

### 2.6. Statistical Analysis

The information was recorded in a database created with the Epi Info™ software (Centers for Disease Control and Prevention, Atlanta, GA, USA), also used for statistical analysis. The qualitative variables were reported as relative frequencies and percentages, while the quantitative variables were presented as mean and standard deviation. The bivariate analysis was performed using Student’s T-distribution, Chi-squared test, using the ANOVA test or non-parametric Kruskall-Wallis test for the bivariate analysis according to the homogeneity or the lack of it in the variance. The Pearson correlation coefficient was used to analyse the linear relationship between two random variables. A difference was declared significant when the probability of type I error was equal to or less than 5%, which was assessed using the Pearson p value, with *p* ≤ 0.05 for that probability value. A linear regression analysis was performed to study the potential association of independent variables (weight, height, age, and BMI) and dependent variables.

### 2.7. Ethical Considerations

This study was approved by the Ethics Committee for Research with medicinal products of the Health Areas of León and Bierzo, with registration number: 1874 (24/04/2018). Principles of informed consent and confidentiality were observed during data collection. The students were assured that the fact that they did or did not participate would in no way affect their academic progress. Necessary permissions were received from the directorate of the Faculty. 

## 3. Results

### 3.1. Socio-Demographic and Anthropometric Characteristics of the Participants

Information was gathered on 112 students. There was a 56% student participation (112/200): 31.3% (35/112) were from the Physiotherapy Degree; 36.6% (41/112) and 32.1% (36/112) were from the Nursing Degree of the campuses of Ponferrada and León, respectively. The age average among the Nursing (León and Ponferrada) and Physiotherapy students was 20.8 years (standard deviation (SD) = 3.2; 18 as a minimum and a maximum of 37). In total, 25.9% of the participants were men and 74.11% were women. They had an average BMI of 22.4 (SD = 2.9; 16.7 as a minimum and a maximum of 30.9) kg/m^2^. No statistical differences were found between the groups analysed except in their height, where the Physiotherapy students presented higher values (*p* < 0.01).

### 3.2. Knowledge 

Data were obtained from 112 students, of which 11.6% (13/112) had been trained in CPR in the two previous years for the completion of the action taken. Knowledge improvement was significant, with a mean of 5.9 out of 10 score (SD = 2) in the pre-test and of 9.6 (SD = 0.6) in the post-test (*p* < 0.001). The stated action improved the post-test scores in a statistically significant way, both by qualification (*p* < 0.01) and by university campus (*p* < 0.01).

**Figure 1:**
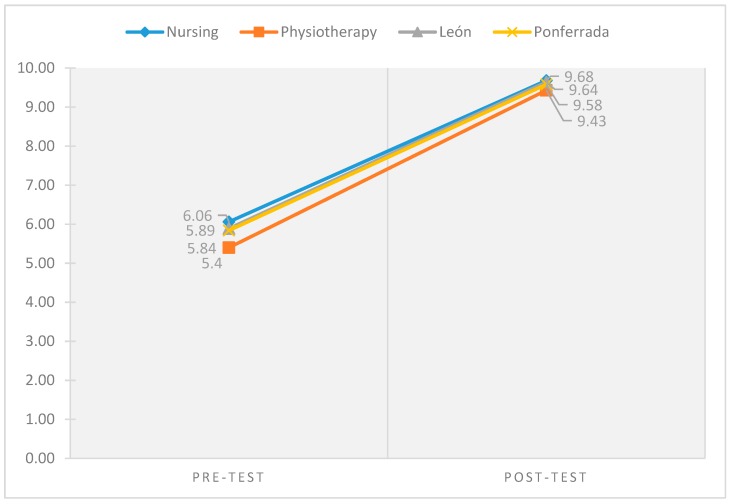
Knowledge improvement by degree and campus.

Figure 1 describes the increase of knowledge stratified by degree (Nursing and Physiotherapy) and by campus (León and Ponferrada).

**Figure 2:**
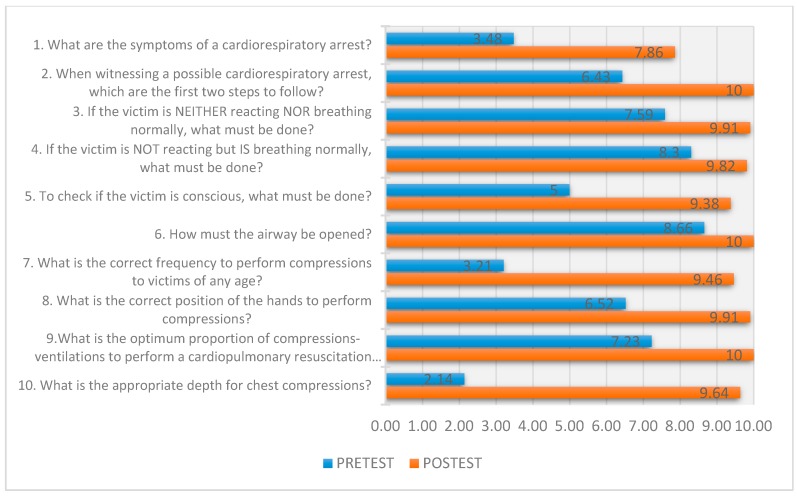
Pre-test and post-test global marks.

Figure 2 presents the mean global scores of both degrees in both campuses obtained by the students in each of the knowledge questions evaluated before and after the action. There were major differences regarding the questions ‘What is the appropriate depth for chest compressions?’ and ‘What is the correct frequency to perform compressions on victims of any age?’ with a difference of 7.5 and 6.3 points, respectively. There was a significant improvement in the results obtained in all questions.

### 3.3. Subjective Evaluation of the Sequence of BLS and Use of AED

Table 2 shows the results obtained by participants in the subjective evaluation of the BLS sequence. The outcomes obtained in most of the items were higher than 75%. The completion of the head tilt/chin lift manoeuvre (89.3%; 100/112), the maintenance of the head tilt/chin lift manoeuvre (84.8%; 95/112), the assessment of the presence of breathing for 10 s (80.4%; 90/112), the proper rhythm of compressions (76.8%; 86/112), the correct opening of the airway (75%; 84/112), nose clamping (83.9%; 94/112), and the insufflation of air for a minute (86.6%; 97/112) were the items with scores lower than 90%. As for the AED, the values obtained were over 90% in all the studied parameters: turn AED on (98.2%; 100/112), follow instructions (94.6%; 106/112), properly placed pads (96.4%; 108/112), performs compressions while charging (95.5%; 205/112), performs minimal interruptions (90.2%; 101/112), and continues the CPR after the electrical shock (97.3%; 109/112).

### 3.4. Objective Assessment of the Quality of CPR

#### 3.4.1. Quality of Compressions

Information on 112 students from the Nursing and Physiotherapy degrees was gathered at the campuses of León and Ponferrada. The mean number of compressions (TNC) in two minutes was 170.7 (SD = 21.9), reaching mean values of 74.7% (SD = 6.8%) for continuous compression (ACC), with the correct position of the hands being 98.8% of the cases.

The mean depth (MD) obtained was 44.9 mm, with a minimum of 22 mm and a maximum depth of 69 mm. The mean percentage of compressions (MPC) performed to the proper depth was 35.6% (SD = 27), while the mean percentage of correct decompression (PCD) was 78.7% (SD = 38.9). In total, 53.2% (SD = 34.1) of the compressions were performed at a proper rhythm (PCPR), obtaining 114.3 (SD = 12.7) as the mean value of compressions (mean rate) per minute performed by the participants.

A significant correlation was found between the mean depth of compressions and the weight (*r* = 0.5), height (*r* = 0.4), and BMI (*r* = 0.4); between the percentage of correct compression and weight (*r* = 0.5) and BMI (*r* = 0.4); between the percentage of quality of CPR (*r* = 0.4) and weight and, finally, between the percentage of compressions at a proper rhythm and weight (*r* = 0.8) (*p* < 0.0001 in all cases).

The percentage of CPR quality presented a statistical relationship with the weight (*b* = 0.9; *p* < 0.001), height (*b* = 0.9; *p* = 0.001), and age (*b* = 2.1; *p* = 0.008). Compression depth mean was also statistically related to weight (*b* = 0.4; *p* < 0.001), height (*b* = 0.4; *p* < 0.001), and age (*b* = 0.7; *p* < 0.001) and, in the same way, the correct depth percentage showed a relationship with weight (*b* = 1.7; *p* < 0.001), height (*b* = 1.5; *p* < 0.001), and age (*b* = 2.9; *p* = 0.013).

#### 3.4.2. Quality of the Ventilations

Information was gathered on administered air volume of 91 participants. The mean volume (MV) reached was 454.9 (SD = 296.3) ml per ventilation. In total, 15.5% (SD = 15.7) ventilations were performed at an appropriate volume (AVV) and students had a mean of 3.2 (SD = 1.9) ventilations per minute (Mean Ratio).

The percentage of students who carried out ventilations with quality volume (ranging between 500 and 600) was 39.6% (36/91). No statistically significant differences by sex and degree were found.

#### 3.4.3. Quality of CPR

The global percentage of quality CPR (QCPR) ranged from the 57.6% (SD = 26.3) on average, with 97% being the highest value obtained. The percentage of students who carried out quality compressions (over 70% in relation to the QCPR) was 31% (45/112). Statistically significant differences by sex and degree were found; male students performed more quality compressions than women (69% vs. 30.1%; *p* < 0.001) (Odds Ratio (OR): 5.2; 95% CI: 2.1–12.9). The same happened with the Physiotherapy students in relation to the Nursing students (54.3% vs. 33.8%; *p* = 0.04) (OR = 2.3; 95% CI: 1–5.3).

Table 3 describes the values obtained in the objective evaluation for each variable analysed in relation to the compressions and ventilations, stratified by sex and degree, respectively. No statistically significant differences in relation to the university campuses were found.

Men performed better than women in overall quality CPR, percentage of correct decompressions, in ventilation and ventilations per minute. Physiotherapy students achieved better results in ventilation, mean volume of ventilations and ratio of ventilations per minute than nursing students.

### 3.5. Difference between Objective Evaluation and Subjective Evaluation

The statistical differences between the items which coincided in the objective and subjective assessment were analysed. Regarding the position of the hands and the compression/decompression, no statistical differences were found (*p* = 0.4 and *p* = 0.7, respectively).

### 3.6. Exertion Made during CPR

Information was gathered on 112 participants. The mean overall exertion was described as ‘hard’ (mean 5.4; SD = 1.7). No statistical differences by degree, campus and sex were found. However, Nursing students valued the exertion as ‘hard’ (mean of 5.7; SD = 1.6), while Physiotherapy students described it as ‘somewhat hard’ (mean of 4.5; SD = 1.7).

## 4. Discussion

This paper has analysed the effect of an educational action in CPR on Health Sciences students of a Spanish University. For the determination of the duration of the action, the results presented by Hernández-Padilla et al. and Aqel et al. were considered, in which they stated this timing as adequate [8,12]. 

As for the duration of the theoretical presentation, the times used by the authors of the work consulted were taken into account, 42 minutes being the shortest duration used by Qi Li et al. [17]. Eventually, the proposal made by the authors of this work, who state that 15 min is enough to acquire the necessary knowledge of CPR, was put into practice. Five minutes per student were estimated as suitable for the acquisition of practical skills, as other studies have pointed out [18,19].

As for knowledge, these results suggest that a theoretical exposure of 15 minutes using a presentation with slides and the subsequent practice of skills was enough to dramatically increase CPR knowledge, obtaining outstanding mean rates as opposed to the mean rate obtained prior to the action. This timing reveals that, with a brief theoretical exposure, good results could be achieved, as opposed to the time used by other authors to date [2,3,10,17], while bearing in mind the recommendation of the ERC on not including theoretical lessons [20]. 

Regarding the relationship between the data obtained in the objective and subjective evaluations, no statistical differences for the position of the hands or the relationship between compression and decompression were found, which suggests that the subjective evaluation by staff trained in CPR is meant to be a reliable measuring instrument.

Nursing students rated the exertion after two minutes of CPR as ‘hard’, while Physiotherapy students qualified it as ‘somewhat hard’, according to the Borg Scale of Perceived Exertion. Our results are higher than those found in professionals who are assumed to be in better physical condition [14,21]. Although no statistical differences were found, this could be due to the fact that Physiotherapy students showed a higher average weight, height and BMI in relation to Nursing students, so their perception of the exertion was lower.

In terms of the quality of compressions, a mean of 85.4 compressions per minute was obtained, which is slightly below the 100–120 compressions per minute recommended by ERC [1]. The same happened with the mean depth participants reached in the compressions (44.9 mm), slightly less than the 50–60 mm the same sources propose. Only 35.6% of students reached the right depth, and 78.7% of the same performed a complete decompression of the chest. Given that the depth of the compression is a value that depends on factors such as weight or height and cannot be completely modified, special importance was placed on the insistence on correct decompression of the victim’s chest as anyone can properly perform it and is one of the CPR quality parameters.

The ERC establishes the appropriate volume to be administered in the ventilation between 500 and 600 cm [1]. The mean volume delivered was 454.9 cm^3^, so they cannot be considered as quality ventilations if we take into account that only 15.5% of them were performed at an appropriate volume. Note that only 75% of the students opened the airway to ventilate the victim, which means that one out of four students did not open the airway for ventilation, precluding the entrance of air in the lungs despite nose clamping and insufflating the right air volume.

Based on the results, we cannot determine that the percentage of overall CPR quality was appropriate, since 57.6% was obtained and experts establish a value higher than 70% as a quality CPR [22].

The results suggest that there is a relationship between weight, height and BMI and some of the studied variables, i.e., compression depth, percentage of correct compression, percentage of global CPR quality, and percentage of correct rhythm during the compressions, obtaining better results with higher levels of weight, height and BMI of the participants, as in previous studies [4,23,24,25].

The sex variable was stated as an influential factor in certain parameters of CPR. Men performed better in mean depth, mean percentage of compressions, percentage of overall CPR quality, ventilation and ventilations per minute. We believe that this is due to men’s greater weight, height and BMI than women, establishing these characteristics as determinants for the performance of quality CPR. 

The action was effective regarding training in the use of the AED, with 90% of the participants obtaining the necessary knowledge and skills. Based on the results, it would be advisable to include this type of training in all courses on BLS and extend this knowledge to the greatest number of people possible, since its use significantly increases the percentage of survival after a CRA and these devices are progressively being implemented in public places.

From the data obtained we can infer that the educational action has been positive in terms of knowledge but has not been effective regarding the practical skills achieved in CPR, as the results are significantly below the values that clinical practice guidelines recommend. We understand that this may be caused by the limited time for the practical training of CPR, the large number of students for each session, or the training method used. As the authors of the work consulted show, through other methods such as self-directed learning, high-fidelity simulation, feedback after surgery or video-guided practice, a more effective CPR training can be offered to Health Sciences university students [8,12,17].

### Limitations of the Study

The participants’ performance was evaluated through a simulation with a manikin. Therefore, the findings may not be applicable to a real cardiorespiratory arrest, during which fear, or other psychological factors may limit the performance.

On the other hand, the study participants were all Health Sciences university students who attended the course on a voluntary basis, so a high motivation and interest in the subject are assumed; as a consequence, the results may not be representative of the general population.

These limitations may be controlled in future studies through the development of Randomised Controlled Trials in various university centres with different training methodologies.

## 5. Conclusions

Training in CPR based on fifteen minutes of theoretical exposure along with practice oriented to the acquisition of skills with feedback was enough to obtain good results in relation to the acquisition of knowledge of BLS, use of AED and skills acquisition in CPR. This study has found a relationship between sex, weight, height, and BMI and the completion of quality CPR, these being variables that allow quality parameters. 

A training method based on a very brief training in CPR through the feedback offered by the manikin and that given by monitors is not sufficient to achieve quality values in the completion of the action. 

The authors of the work have, as a proposal for future lines of research, to investigate the time interval after which it is necessary to carry out knowledge and skills retraining in cardiopulmonary resuscitation. In addition, it is appropriate to carry out a randomised clinical trial in different populations of students with different CPR training methods. 

## Figures and Tables

**Table 1:** List of variables resulting from the questionnaire and the CPR.

Resulting from the Questionnaire	Linked to the CPR
OHCA symptom identification. First steps to take in an OHCA. Actions to take if the victim is neither reacting nor breathing. Actions to take if the victim is not reacting but is breathing. Consciousness checking. Airway opening method. Correct frequency of chest compressions. Correct position of hands. Correct proportion of compressions-ventilations Correct depth of chest compression.	Sequence of CPR. Correct position of hands (HP). Total number of compressions (TNC). Continuous compression percentage (CCP). Reached average depth (AD). Compression percentage with the appropriate depth (CDP). Correct decompression percentage (CDcP). Proper rhythm compressions percentage (PCPR). CPR global quality percentage (QCPR). Average rhythm of compressions (Average Rhythm). Compression-decompression ratio (Average Ratio). Exertion applied.

CPR: Cardiopulmonary Resuscitation. OHCA: Out-of-hospital cardiorespiratory arrest.

**Table 2:** BLS sequence subjective evaluation.

Performance	*n*	%	95% CI	
Safe approach	111	99.11	95.13	99.98
Checks consciousness	107	95.54	89.89	98.53
Asks and shouts	108	96.43	91.11	99.02
Shakes it	107	95.54	89.89	98.53
Opens airway	110	98.21	93.70	99.78
Head tilt/chin lift	100	89.29	82.03	94.34
Checks breathing	111	91.11	95.13	99.98
Holds Head tilt/chin lift	95	84.82	76.81	90.90
See-Hear-Feel	110	98.21	93.70	99.78
Assesses in 10 s	90	80.36	71.78	87.26
Calls 112	109	97.32	92.37	99.44
Asks for AED	108	96.43	91.11	99.02
Performs 30:2	110	98.21	93.70	99.78
Starts with compressions	112	100.00	96.76	100.00
Correct position of hands	106	94.64	88.70	98.01
Placement on the victim’s vertical	112	100.00	96.76	100.00
Appropriate compression/decompression	100	89.29	82.03	94.34
Appropriate rhythm	86	76.79	67.86	82.24
Opens Airway	84	75.00	65.93	82.70
Clamps nose	94	83.93	75.79	90.19
Insufflates 1 s	97	86.61	78.87	92.31
Turns AED on	110	98.21	93.70	99.78
Follows instructions	106	94.64	88.70	98.01
Places pads	108	96.43	91.11	99.02
Compressions while charging	107	95.54	89.89	98.53
Minimal interruptions	101	90.18	83.11	94.99
Continues CPR	109	97.32	92.37	99.44

AED: Automatic External Defibrillator. CI: Confidence interval.

**Table 3:** Variables distribution of the Skill Reporter organised by Sex and Degree.

Manoeuvre	Sex					Degree				
		*n*	Mean	SD	*p*-Value		*n*	Mean	SD	*p*-Value
**QCPR**	Male	29	75.86	18.11	0	Nursing	77	55.18	24.914	0.159
	Female	83	51.16	25.836		Physiotherapy	35	62.77	28.94	
**CCP**	Male	29	73.17	4.4	0.176	Nursing	77	74.65	7.572	0.996
	Female	83	75.17	7.441		Physiotherapy	35	74.66	4.881	
**HP**	Male	29	98.66	4.002	0.952	Nursing	77	98.19	13.037	0.228
	Female	83	98.8	12.37		Physiotherapy	35	100	0	
**TNC**	Male	29	166.21	16.862	0.197	Nursing	77	169.09	21.293	0.24
	Female	83	172.31	23.249		Physiotherapy	35	174.34	22.987	
**MD**	Male	29	51.9	7.267	0	Nursing	77	44.18	10.263	0.268
	Female	83	42.43	9.572		Physiotherapy	35	46.43	9.056	
**CDcP**	Male	29	64.07	33.515	0.005	Nursing	77	79.12	26.905	0.812
	Female	83	83.82	22.37		Physiotherapy	35	77.8	27.554	
**CDP**	Male	29	63.93	36.206	0	Nursing	77	32.62	39.75	0.236
	Female	83	25.66	34.86		Physiotherapy	35	42.06	36.623	
**CRC**	Male	29	56.66	30.72	0.532	Nursing	77	50.74	35.865	0.22
	Female	83	52.04	35.266		Physiotherapy	35	58.71	29.492	
**RM**	Male	29	114.31	12.613	0.98	Nursing	77	113.12	13.023	0.159
	Female	83	114.24	12.813		Physiotherapy	35	116.77	11.763	
**Ratio**	Male	29	0.7366	0.20391	0.237	Nursing	77	0.7157	0.20697	0.106
	Female	83	0.6751	0.25086		Physiotherapy	35	0.6366	0.29702	
**Ventilations**	Male	29	8.17	2.674	0.006	Nursing	77	6.21	3.975	0.026
	Female	83	6.27	4.159		Physiotherapy	35	7.97	3.519	
**MV**	Male	28	374.82	273.641	0.087	Nursing	60	499.87	309.737	0.044
	Female	63	490.43	302.05		Physiotherapy	31	367.74	252.999	
**VEV**	Male	19	27.05	29.975	0.718	Nursing	38	29.58	32.006	0.902
	Female	40	30.23	32.036		Physiotherapy	21	28.52	30.341	
**AVV**	Male	22	15.45	17.717	0.977	Nursing	35	14.8	13.96	0.673
	Female	38	15.58	14.733		Physiotherapy	25	16.56	18.203	
**NAVV**	Male	22	16.55	15.396	0.674	Nursing	50	15.68	20.227	0.168
	Female	55	18.91	24.309		Physiotherapy	27	22.96	24.806	
**MEANRATIO**	Male	29	3.93	1.387	0.007	Nursing	77	2.94	1.935	0.018
	Female	83	2.98	2.036		Physiotherapy	35	3.86	1.785	

QCPR: Global quality of CPR; CCP: Continuous compression percentage; HP: Hands position; TNC: Total number of compressions; MD: Mean depth; CDcP: Correct decompression percentage; CDP: Correct depth percentage; CRC: Correct rhythm compressions; RM: Rhythm of compressions per minute; Ratio; Compressions-ventilations base 1 relation; Ventilations: Total number of ventilations in 2 min; MV: Mean volume applied. VEV: Ventilations exceeding the maximum volume; AVV: Appropriate volume ventilations; NAVV: Non-appropriate volume ventilations; MEANRATIO: Number of ventilations per minute.
